# Preparation and Characterization of Polyvinyl Alcohol (PVA)/Carbonized Waste Rubber Biocomposite Films

**DOI:** 10.3390/polym16081050

**Published:** 2024-04-11

**Authors:** Mustafa Zor, Ferhat Şen, Zeki Candan, Evgeni Ivanov, Todor Batakliev, Vladimir Georgiev, Dzhihan Menseidov

**Affiliations:** 1 Department of Nanotechnology Engineering, Zonguldak Bülent Ecevit University, 67100 Zonguldak, Türkiye; mustafa.zor@beun.edu.tr; 2 Biomaterials and Nanotechnology Research Group & BioNanoTeam, 34473 İstanbul, Türkiye; zekic@istanbul.edu.tr; 3 Department of Forest Industrial Engineering, İstanbul University-Cerrahpasa, 34473 İstanbul, Türkiye; 4 Institute of Mechanics, Bulgarian Academy of Sciences, 1113 Sofia, Bulgaria; ivanov_evgeni@imbm.bas.bg (E.I.); todorbat@gmail.com (T.B.); vgeorgiev@imbm.bas.bg (V.G.); menseidov@imbm.bas.bg (D.M.); 5 Department of Telecommunications, University of Telecommunication and Post, 1700 Sofia, Bulgaria

**Keywords:** polyvinyl alcohol, carbonized waste rubber, biocomposites

## Abstract

The technological properties of composite materials (thermal, strength, rheology, electrical and morphology) are very important parameters for high-performance applications. In this study, we aimed to improve the properties of PVA by using carbon materials obtained by the pyrolysis of waste tires, with the aim of recycling them instead of disposing of them. For this purpose, PVA biocomposite films containing carbonized waste rubber at different rates were prepared. The thermal properties of the prepared biocomposite films were examined via thermal gravimetric analysis (TGA) and differential scanning calorimetry (DSC) methods. While rheological measurements were carried out with a rheometer, bulk conductivities were measured with a pico-ammeter. In addition, the morphology of biocomposite films was determined via field emission scanning electron microscopy. The nanomechanical properties of biocomposite film was investigated via XPM analyses. According to the rheological measurements and nanoindentation hardness results, it is understood that as the amount of carbonized waste rubber increases, flexibility decreases and harder and brittle structures are observed in biocomposite films. The electrical measurement results showed that electrical conductivity increased as the amount of carbonized waste rubber increased. When all the results obtained were evaluated, it could be concluded that biocomposite films obtained by increasing the electrical conductivity and hardness of PVA can be used in the electronics industry.

## 1. Introduction

Biocomposites, which contain a wide variety of organic and/or inorganic components such as natural and synthetic polymers, polysaccharides, proteins, sugars, ceramics, metals and nanocarbons, are defined as biocompatible and/or environmentally friendly composites [1]. As a structure, it can be seen in the form of films, membranes, molds, coatings, fibers, particles or foams. In addition to increasing the functionality of mechanical properties in many materials, biocomposites also take their place in the field of environmentally friendly composites in medical fields such as sensors, tissue engineering and implants [2]. While developments in technology in recent years, depending on consumer demands and expectations, continue to increase the demand for global resources, they can cause serious problems in terms of ease of access to materials and environmental sustainability. This situation has shown that there is intense interest in biofiber and biocomposites. It is known that with the increasing acceleration of the latest technology, bio-based engineering materials are being used instead of petro-derived materials, and serious investments are being explored to increase the competition in the global market. It seems that there are studies that tend to transform plastics into high-value-added materials by mixing and separating them in the simplest way instead of disposing of them as waste materials in nature [3,4]. The disposal of non-degradable plastic materials causes environmental problems, which seriously encourages environmental sustainability issues [5,6]. Some of the most commonly found biodegradable polymers are poly(lactic acid) (PLA), polyvinyl alcohol (PVA), polyhydroxybutyrate (PHB) and poly(butylene adipate-co-terephthalate) (PBAT). PVA is a hydrophilic synthetic biodegradable polymer obtained by the polymerization of vinyl acetate monomer to polyvinyl acetate (PVAc) and the hydrolysis of acetate groups [7]. The degree of this hydrolysis varies depending on the saponification reaction [8]. In addition to being a water-soluble polar polymer, PVA has high water resistance and optical transparency [9,10]. Due to this feature, it is used in packaging and as an optical polarizer. In addition, due to the ease of recycling, it is used in binding, coating or adhesives; due to its high chemical and thermal resistance and low cost, it is preferred in many industries such as the textile industry, the paper industry and food packaging [11,12,13,14]. The advantage of PVA in composite production is that it is non-toxic and a very durable polymer [15]. On the other hand, its disadvantages are its high costs, high water absorption power and low mechanical strength, which limit all kinds of engineering applications of PVA. In this case, it is possible to convert it into a high-value-added product when combined with low-cost biodegradable materials such as cellulose, nanocellulose, nanoclay and biowaste materials [16,17,18].

According to the literature, the mechanical properties of PVA vary according to the molecular weight (200,000–400,000 g/mol) and degree of hydrolysis (80–99%, i.e., 84.2–89% (partial hydrolysis), 92.2–96% (moderate hydrolysis) and 98−99%). Tang and Alavi [19] reported that to further improve the properties of PVA (such as mechanical properties, sensitivity to moisture), some physical or chemical methods such as cross-linking and addition of new nanoparticles can be applied, and materials with a wide range of property profiles have been developed with these new techniques. In another study reported that PVA is used especially in biomedical and pharmaceutical applications [20], construction and building materials [21], and optical sensors [22] due to its biocompatibility, reliability, good hydrogel formation, and high swelling properties. It has been reported to be used in various fields such as packaging [23]. Zor et al. [24] used waste tire pyrolysis oil obtained as a result of pyrolysis and evaluated this material as a reinforcement element in nanocellulose-based nanocomposite films. According to the results, they revealed that nanocellulose-based pyrolysis oil nanocomposite films with superior thermal properties and structural compatibility demonstrated by characteristic results can be used in future industrial applications such as pharmaceuticals, coatings and green packaging. Verma et al. [25] produced and characterized composites using epoxy resin with pyrolysis oil obtained from the pyrolysis of tire waste. The results showed that composites with lower density and higher tensile strength than pure epoxy can be produced, and the produced composites can be used in the automotive industry.

It is known that large amounts of worn synthetic and natural rubber are thrown out every year, posing a threat to human life and the environmental [26]. The production of waste is an outcome of urbanization and population growth along with economic development. It has been determined that 2.01 billion tons of municipal waste have been generated since 2016, and this figure is expected to reach 3.3 billion tons by 2050 [27]. To bring these wastes into economic viability, they are produced in many composite forms to provide high strength/hardness for reinforcement purposes. For example, rubber sheets are reinforced with various materials such as Kevlar, glass cloth, steel, aramid, nylon and polyester. Sustainable environmental policies are considered in rubber waste management for industrial exploitation. This situation provides great opportunities for creating a circular economy [28].

Based on the previous discussion, PVA is compatible with other polymers, so this study will evaluate its compatibility with PVA polymers instead of disposing of carbonized waste tires. In this study, we aimed to improve the properties of PVA by using carbon materials obtained via the pyrolysis of waste tires, with the aim of recycling them instead of disposing of them. For this purpose, PVA biocomposite films containing carbonized waste rubber at different rates were prepared. Thermal, rheological, electrical, morphological and nanomechanical properties of the prepared biocomposite films were examined.

## 2. Materials and Methods

### 2.1. Materials

**Figure 1:**
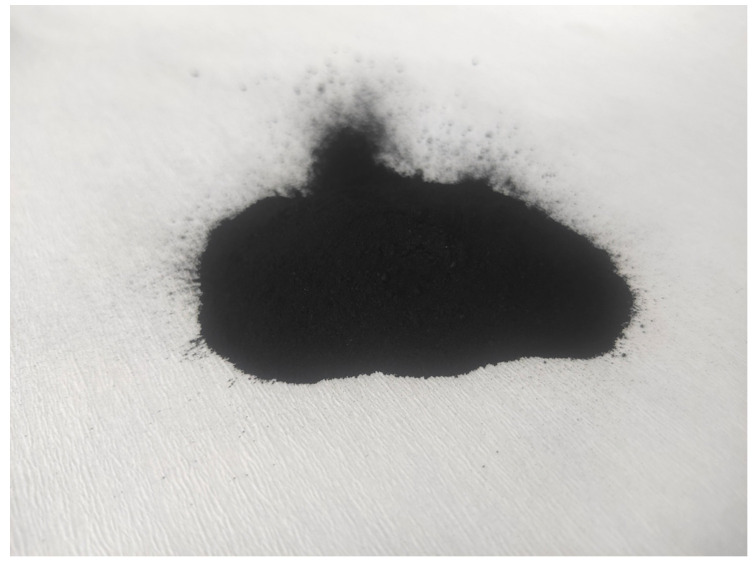
Carbonized waste tire powder.

Waste tires were subjected to pyrolysis under high pressure, and carbonized waste rubber was produced from ZBB GmbH (Mannheim, Germany). The obtained carbon materials used in the study are shown in Figure 1. It consists of black powder particles with sizes of 0–15 µm. PVA was purchased from Sigma-Aldrich (St. Louis, MO, USA).

### 2.2. Preparation of the Biocomposite Films

Moreover, 3 g of PVA and carbonized waste rubber (0%, 0.5% and 1% by mass of PVA) were weighed in a beaker. Furthermore, 100 mL of distilled water was added to the beaker. The beaker was kept at 80 °C for 2 h to completely dissolve the PVA. The beaker was kept in an ultrasonic bath for 30 min to ensure the homogeneous distribution of carbonized waste rubber in the mixture. The mixture was poured into Petri dishes and dried in an oven at 40 °C for 48 h. Biocomposite films were obtained from the Petri dish. Samples were coded as PVA, PVA0.5CR and PVA1CR. PVA represents the sample without carbonized waste rubber, and PVA0.5CR and PVA1CR represent the samples containing 0.5% and 1% carbonized waste rubber.

### 2.3. Characterization and Measurements

TGA Q50 (TA Instruments, New Castle, DE, USA) was applied for the thermo-gravimetric analysis (TGA) measurements. Thermogravimetric analysis (TGA) was made to assess the composite thermal stability defined by the characteristic decomposition temperatures of the samples. TGA was carried out in a N_2_ atmosphere with a heating temperature of 25 °C per minute in the range of 40–600 °C. DSC Q20 (TA Instruments) was used for the DSC measurements. DSC analysis was carried out in a N_2_ atmosphere with a heating temperature of 10 °C per minute in the range of 50–250 °C.

The rheological measurements were carried out with an AR-G2 rheometer (TA Instruments, New Castle, DE, USA) using electrical heated parallel plate geometry with a diameter of 25 mm. Both oscillatory and shear flow test modes were applied, where the dynamic modulus (G′, G″) were determined with varying frequency or shear rate, respectively, in the range 0.01–100 s^−1^. The test samples were hot-pressed disks with a diameter of 20 mm and thickness of 2 mm. Rheology Advantage Data Analysis V5.0.7 software was used for performing the experiments and rheological calculations [29].

A pico-ammeter (Keithley 6517B Electrometer/High Resistance Meter, Keithley Instruments Inc., Beaverton, OR, USA) was used to measure the bulk conductivity. Three-disc samples with a diameter of 22 mm and thickness of 0.33 mm of each composition were tested. The test was conducted at room temperature of 20 °C with direct readings of the flushing current between two adjacent metallized zones. During the test, the electrical resistivity of the material in Ohm was measured [29].

Visualizations were made by using Tabletop SEM SH-4000M (Hirox, Tokyo, Japan) in order to observe the morphology of the biocomposite films. XPM (fast indentation or accelerated property mapping) was performed on the Hysitron TI 980 instrument (Bruker, MN, USA) by using a 2D transducer assembly (both normal and lateral force) equipped with a Berkovich probe and applying peak force of 1000 μN. In general, the XPM testing method is performed in the same way as the standard nanoindentation technique but is faster as it reduces the time of the functional segments.

## 3. Results

### 3.1. Thermal Properties of Biocomposite Films

**Figure 2:**
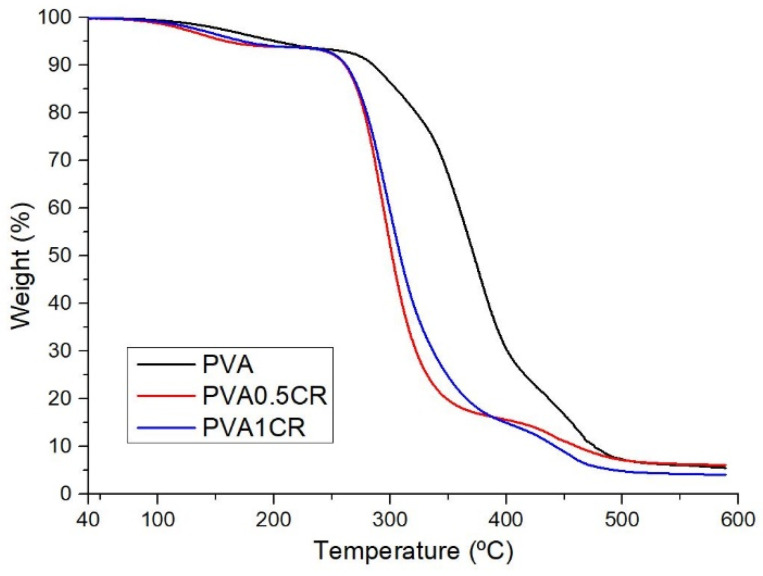
TGA of biocomposite films.

The thermal stability of the biocomposite films was examined via the TGA technique. It is seen that the samples have their first mass loss between 160 and 203 °C. This mass loss is attributed to the removal of intermolecular water. The main decomposition temperatures of the samples are in the range of 309–372 °C (Figure 2). The results obtained show that thermal stability decreases with increasing amounts of carbonized waste rubber due to the disruption of cross-linking or weak interactions within the PVA molecule caused by the carbonized waste rubber additive. On the other hand, it is seen that the char yield of the PVA0.5CR sample is higher than that of PVA, but the char yield of the PVA1CR sample is lower.

**Figure 3:**
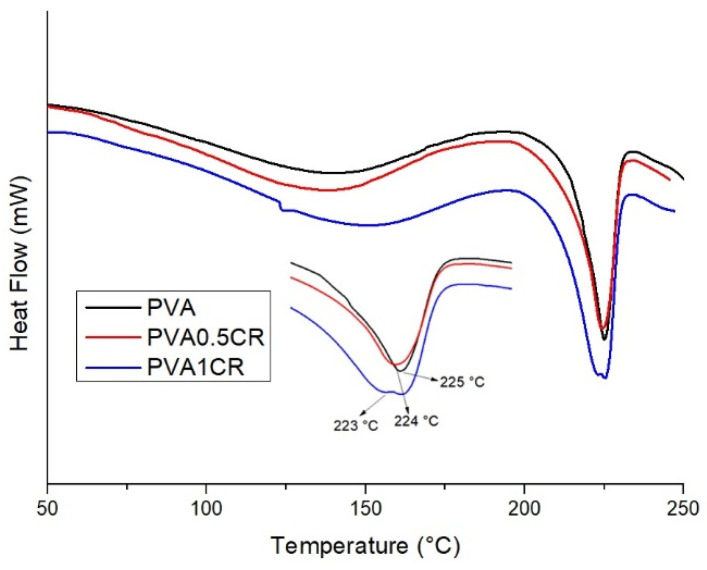
DSC of biocomposite films.

Melting temperatures (Tm) of the samples were examined via the DSC technique (Table 1). When the results obtained were examined, it was observed that there was no significant change in the melting temperatures. While the melting temperature of PVA was 225 °C, the melting temperature of PVA1CR was determined as 223 °C (Figure 3).

### 3.2. Rheological Properties of Biocomposite Films

To elucidate the structure–property relationships in polymer composites, comprehending their rheological behavior in the melt state is crucial. Melt rheology measurements, as observed in our data, offer insights into the behavior of these materials on a macroscopic scale, which is vital for understanding how modifications in composition affect material properties. In the rheological measurements, dynamic drag test was applied to these samples, which were prepared to define the strain dependence of the viscoelastic properties of the samples and to determine the linear viscoelastic region. The rheological parameters of the test samples are given in Table 2.

As the frequency value increases in PVA composites, the loss modulus also increases and the composites show more viscous properties. As the frequency value increases in the complex viscosity, the viscosity decreases in the properties. In the storage modulus, on the other hand, as the frequency value increases, the elastic value increases. PVA shows more elastic properties than other composite materials. With the increase in PVA 1% additive, the elastic behavior of the storage modulus is effective. According to the frequency value, while PVA is more elastic, carbonized waste rubber shows less elastic behavior. The viscoelastic behavior was confirmed for all composite as the elastic modulus (G′) was significantly greater than the viscous modulus (G″) for the entire frequency range. Similar results are seen in the study by Raschip et al. [30].

**Figure 4:**
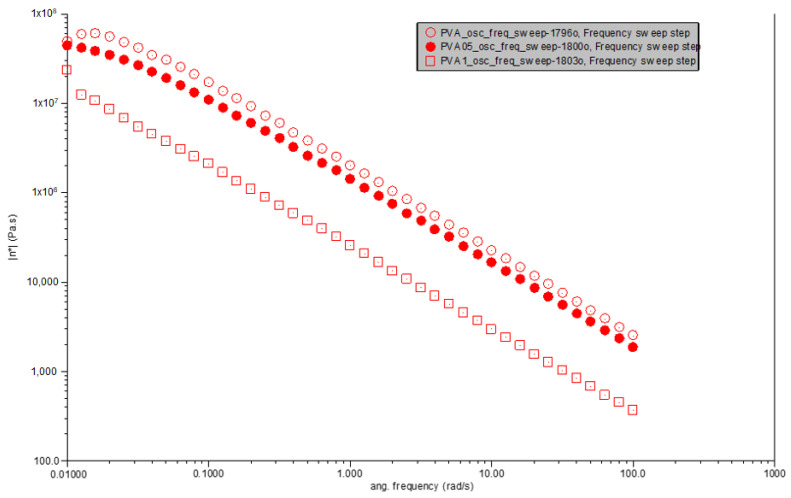
Viscosity vs. angular frequency for PVA, PVA0.5CR and PVA1CR.

Observations from Figure 4 reveal that pure PVA exhibits a decrease in complex viscosity with increasing angular frequency, a behavior indicative of shear-thinning. This phenomenon is characteristic of polymeric materials, where molecular alignment under shear stress leads to a reduction in flow resistance. At the lowest angular frequency (0.01 rad/s), the complex viscosity of pure PVA is observed to be approximately 4.85 × 10^6^ Pa·s, which progressively decreases with increasing angular frequency, underscoring the material’s sensitivity to shear rate.

The introduction of 0.5% carbonized waste rubber to create PVA0.5CR modifies this shear-thinning behavior slightly. At the same angular frequency (0.01 rad/s), the complex viscosity is approximately 4.35 × 10^6^ Pa·s, a value that, while still indicative of shear-thinning, is lower than that of pure PVA. This suggests that the dispersion of carbonized waste rubber within the PVA matrix affects the flow behavior, likely due to the interaction between the rubber particles and the polymer chains, altering the composite’s resistance to flow.

With a further increase in filler content, PVA1CR, containing 1% carbonized waste rubber, continues to demonstrate shear-thinning behavior. The complex viscosity at the lowest angular frequency (0.01 rad/s) is significantly reduced to approximately 2.37 × 10^6^ Pa·s. This reduction in viscosity, compared to both pure PVA and PVA0.5CR, suggests a more pronounced effect of the filler–matrix interaction and the possible formation of a conductive filler network. Such a network could facilitate flow under applied shear, indicating that the presence of carbonized waste rubber not only influences the viscosity but also enhances the material’s ability to deform and flow more readily under stress.

This analysis underscores the influence of carbonized waste rubber content on the rheological properties of PVA-based composites, revealing a clear trend of decreasing complex viscosity with increased filler content. This behavior highlights the potential of such composites for applications requiring materials with adjustable flow properties under varying shear conditions. The incorporation of carbonized waste rubber into PVA distinctly affects the complex viscosity across angular frequencies. While all materials exhibit shear-thinning behavior, the degree of viscosity reduction with increasing angular frequency varies. PVA0.5CR shows a slight decrease in viscosity at lower frequencies compared to pure PVA, suggesting that a small amount of filler enhances the material’s resistance to flow due to the filler–matrix interactions. Conversely, PVA1CR presents a lower viscosity at similar conditions, potentially due to the altered microstructure and enhanced filler networking within the matrix, which may facilitate easier flow under shear.

The comparative analysis reveals that the addition of carbonized waste rubber to PVA significantly influences its rheological behavior, particularly the complex viscosity. The observed trends underscore the complex interplay between filler content, filler–matrix interactions, and the resultant viscoelastic properties. The incorporation of carbonized waste rubber as a filler into polyvinyl alcohol (PVA) significantly alters its rheological properties, particularly by inducing shear thinning behavior and changing this complex viscosity, which reflects the interplay between the filler content and polymer matrix interactions.

**Figure 5:**
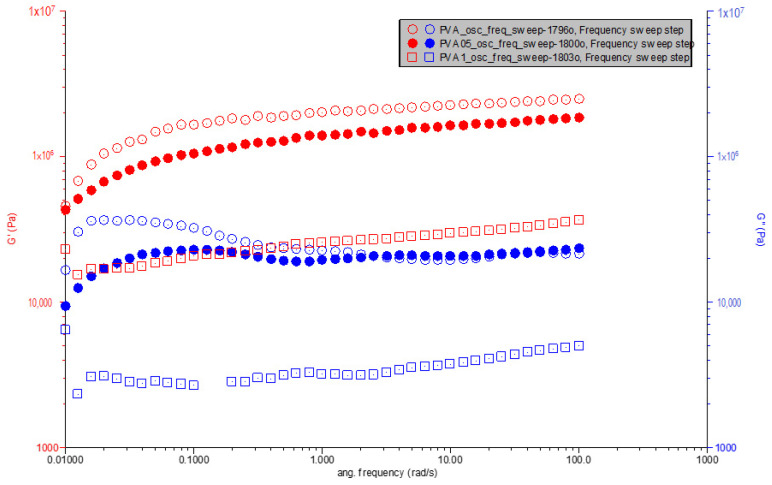
G′ & G″ vs. angular frequency for PVA, PVA0.5CR and PVA1CR.

As we observe in Figure 5, the viscoelastic properties of polymer composites, such as storage modulus (G′) and loss modulus (G″), are pivotal in understanding their behavior under dynamic conditions. This analysis delves into how the incorporation of carbonized waste rubber into polyvinyl alcohol (PVA) influences these properties across a spectrum of angular frequencies. Pure PVA showcases a characteristic increase in both G′ and G″ with angular frequency, highlighting its viscoelastic nature with a pronounced elastic response that is complemented by viscous dissipation. The initial values at the lowest angular frequency for G′ and G″ are observed at approximately 42,480 Pa and 9356 Pa, respectively.

Introducing 0.5% carbonized waste rubber into PVA alters this viscoelastic profile. The modified composite, PVA0.5CR, exhibits initial G′ and G″ values at the lowest angular frequency of approximately 50,530 Pa and 12,370 Pa, respectively. This enhancement in both moduli suggests an increase in the material’s stiffness and its ability to dissipate energy, likely due to the interaction between the rubber particles and the PVA matrix. The presence of the filler seems to facilitate a more pronounced viscoelastic response, indicating effective load transfers and interactions within the composite.

The effect of filler incorporation is further emphasized in PVA with 1% carbonized waste rubber (PVA1CR). This composition shows initial G′ and G″ values at the lowest angular frequency significantly different from both pure PVA and PVA0.5CR, with G′ at approximately 22,870 Pa and G″ at 6392 Pa. The distinct behavior of PVA1CR, especially the lower initial G′ compared to its counterparts, might suggest complex interactions at higher filler loadings, which could affect the composite’s overall stiffness and viscous behavior. Despite the lower initial stiffness, the trend of increasing moduli with frequency is maintained, suggesting that at higher filler contents, the composite’s structure and dynamics might be altered, possibly due to aggregation or phase morphology changes.

The incorporation of carbonized waste rubber into PVA distinctly influences its storage and loss moduli, with the magnitude of these moduli and their rate of change with frequency varying with filler content. The analysis reveals that even small amounts of filler significantly affect the material’s response to dynamic loading, underscoring the complex interplay between filler content, polymer–filler interactions and the resultant composite properties. These findings not only highlight the critical role of filler content in modulating the viscoelastic properties of polymer composites but also provide a foundation for further exploration into the mechanisms governing these effects, aiming at optimizing material formulations for targeted applications.

### 3.3. Electrical Properties of Biocomposite Films

Generally, polymer materials are known to have low electrical conductivity and are widely used in electrical and electronic applications due to these properties. However, in many applications where polymer materials are needed, electrical conductivity is also needed. For this reason, composite materials have been developed by adding materials with high electrical conductivity such as carbon black, carbon fiber, graphite, graphene, metal and metal oxides and carbon nanotubes to polymer materials. The electrical conductivity of polymer materials varies according to the amount of additives and electrical conductivity. The electrical conductivity measurements of the test samples are given in the Table 3.

Upon examining the electrical conductivity of biocomposite films, it is observed that the conductivity enhances with the increment of carbonized waste rubber content. Relative to pure PVA, the conductivity of PVA0.5CR shows an increase of 7.9%, and for PVA1CR, the increase is more pronounced at 27.5%. This trend demonstrates that the addition of carbonized waste rubber effectively boosts the electrical conductivity of the composites, aligning with findings reported in the literature. The observed enhancement in electrical conductivity with the incorporation of carbonized waste rubber into polyvinyl alcohol (PVA) composites not only underscores the potential of waste materials as functional fillers in polymer matrices but also highlights the nuanced interplay between filler content and composite properties. This increase in conductivity, particularly the substantial 27.5% jump observed with the 1% carbonized waste rubber addition, suggests a threshold beyond which the conductive networks within the composite become more efficient at facilitating electron transport. Such behavior is emblematic of the percolation threshold in composite materials, where a minimal amount of conductive filler leads to a disproportionate increase in conductivity, marking a transition from an insulating to a conductive state.

Moreover, this trend of increasing conductivity with higher filler content opens intriguing possibilities for tailoring the electrical properties of polymer composites to specific application needs, ranging from static dissipation surfaces to conductive elements in flexible electronics. The use of carbonized waste rubber adds a layer of environmental sustainability to this technological advancement, transforming waste into a valuable resource for the development of advanced materials. This approach not only aligns with the principles of circular economy but also offers a pragmatic solution to the dual challenges of waste management and the demand for functional materials in the electronics industry.

### 3.4. Morphology Properties of Biocomposite Films

**Figure 6:**
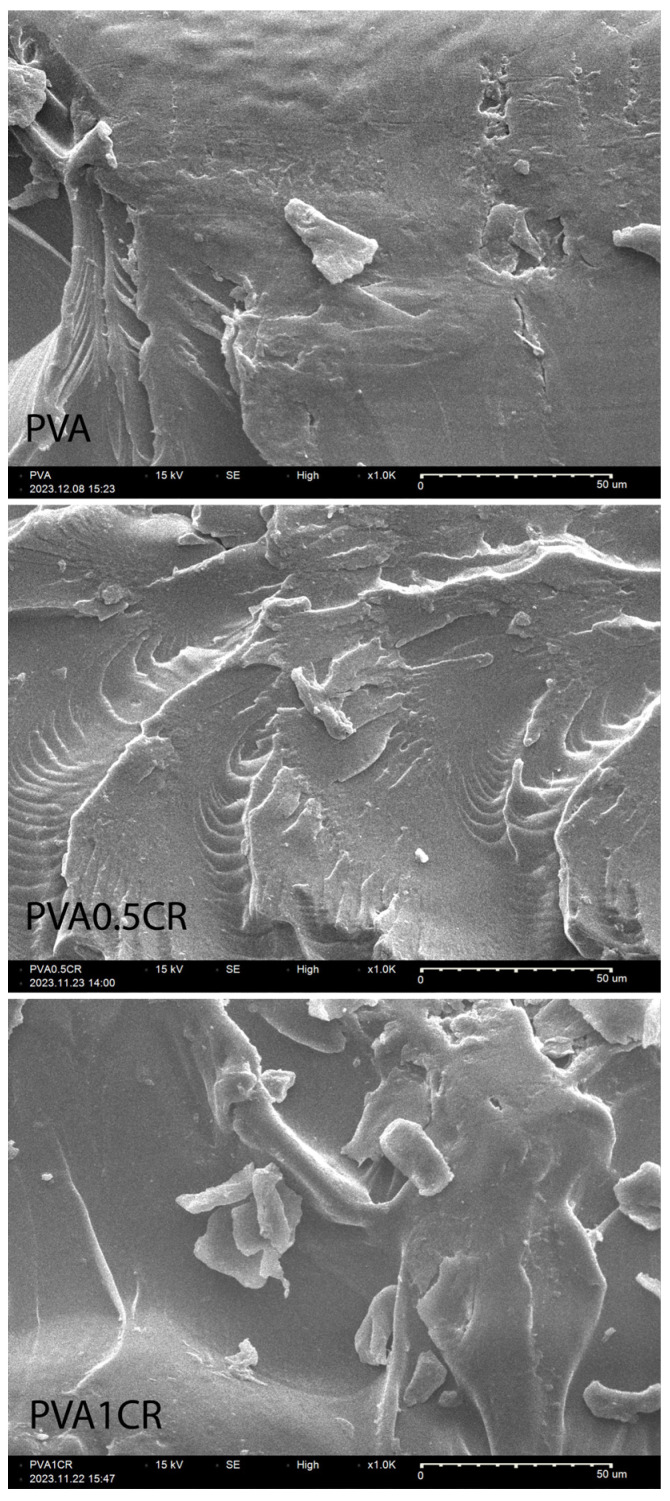
SEM images of biocomposite films.

The morphologies of biocomposite films were examined using the SEM technique. SEM images of the samples at ×1000 magnification are shown in Figure 6. When the SEM images are examined, it is understood that the carbonized waste rubber additive does not clump in the polymer matrix. It has been determined that PVA and carbonized waste rubber are compatible with each other, and homogeneous biocomposite films are obtained.

### 3.5. Nanomechanical Properties of Biocomposite Films

**Figure 7:**
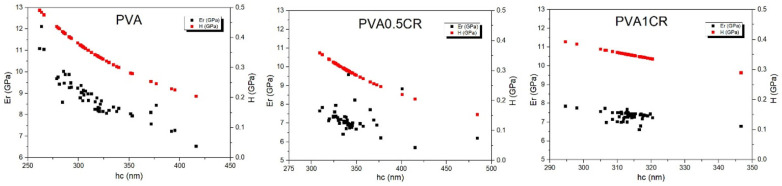
Nanoindentation hardness (H) and reduced elastic modulus (Er) of biocomposite specimens including neat PVA as a reference.

The effective or reduced elastic modulus (Er) and hardness (H) of the samples were determined by an instrumental nanoscale indentation technique according to the Oliver– Pharr method [31] as a principal experimental approach for the surface characterization of the composite films. Nanoindentation hardness (H) and reduced elastic modulus (Er) values are shown in Figure 7. Considering the average H values, it was calculated that the nanohardness of the three materials labeled as PVA, PVA0.5CR and PVA1CR was 297, 354 and 351 MPa, respectively. On the other hand, when the average Er values were examined, the reduced elastic modulus of PVA was found to be 8.84 GP against 7.16 and 7.34 GPa for PVA0.5CR and PVA1CR. As a result, an increase of 18–19% in hardness and a decrease of 17–19% in elastic modulus of the composites were observed with the carbonized waste rubber additive. This nanomechanical behavior of the samples can be explained with the low displacement being in the range of several hundreds of nanometers where rather the plastic properties of the polymer films come from. Regarding the hardness measurement, a stable nanomechanical tendency was recorded for each nanocomposite sample, revealing a gradual decrease in hardness at greater penetration depths. Similar results are seen in the study by Batakliev et al. [32].

**Figure 8:**
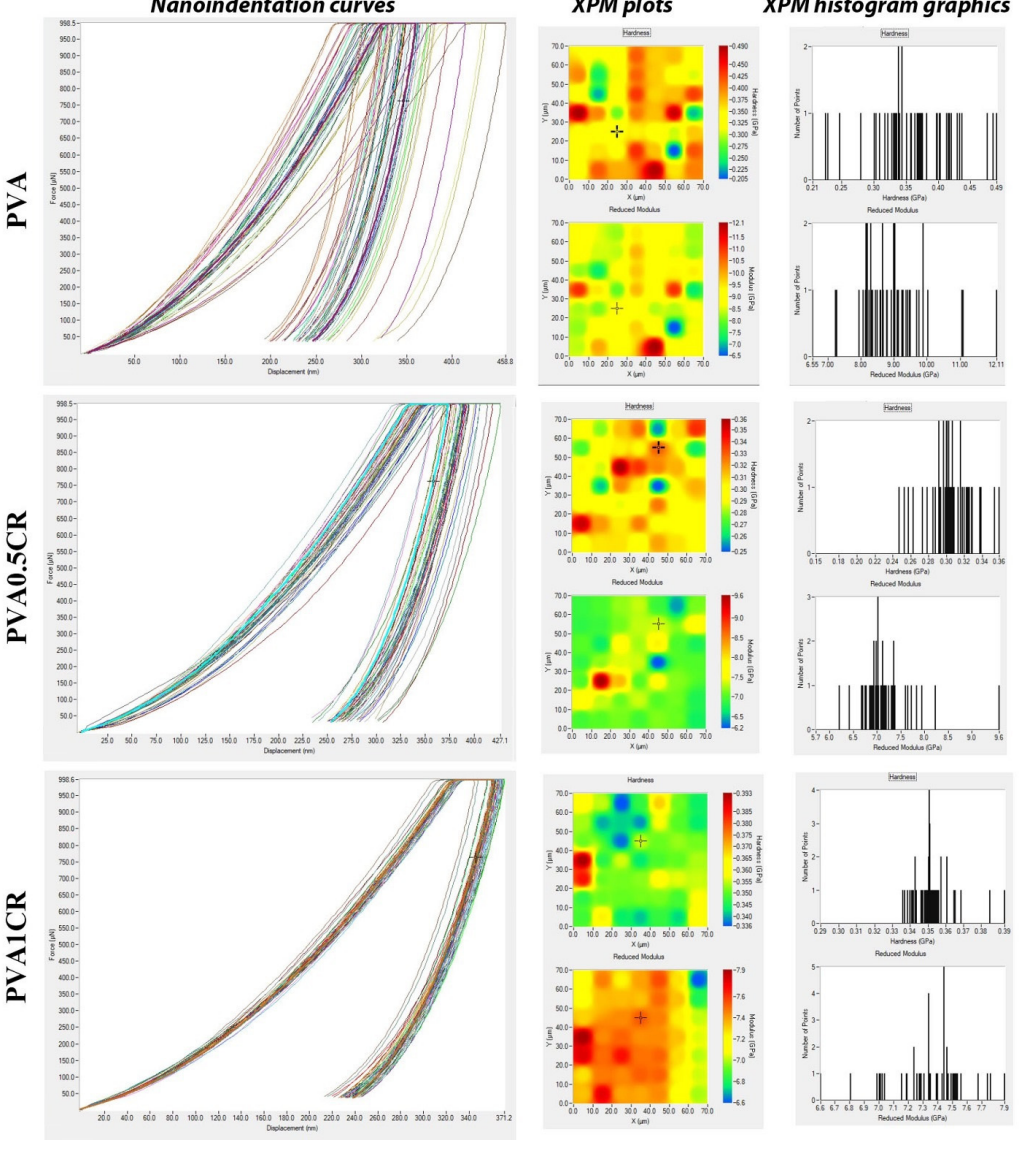
Nanoindentation curves and XPM plots, XPM histogram graphics of biocomposite films.

Nanoindentation curves, XPM plots and XPM histogram graphics of the biocomposite films are shown in Figure 8.

Fast nanoindentation of the modified polymeric materials has been made via the XPM technique, which expands over surface nanomechanical features of different locations in a definite surface area of the composite sample. This technique is the basis of the mapping method, allowing high-speed nanoindentation, that is, mapping the mechanical properties of the local surface area in a fast time [33]. When the XPM plot images are examined, relatively homogeneous distributions in hardness and reduced elastic modulus are observed. This homogeneous distribution is more evident in the reduced elastic modulus of carbonized waste rubber-added composites. On the other hand, in the XPM histogram graphs, it is seen that the nanomechanical properties are in a much shorter range with respect to the composite films. These results are indicative of the carbonized waste rubber contribution to the surface nanomechanical parameters of the new materials. 

## 4. Conclusions

In this study, we aimed to improve the properties of PVA by using carbon materials obtained by the pyrolysis of waste tires, with the aim of recycling them instead of disposing of them. Different ratios of PVA and carbonized waste rubber biocomposite films were successfully obtained, and characterization tests were carried out. The results obtained from the tests can be listed as follows:

- The results obtained from TGA showed that thermal stability decreased as the amount of carbonized waste rubber increased as the carbonized waste rubber additive disrupts cross-linking or reduces weak interactions within the PVA molecule.
- The results obtained from DSC showed that there was no significant change in the melting temperature of biocomposite films as the amount of carbonized waste rubber increased.
- From the rheological measurements, it was understood that as the amount of carbonized waste rubber increased, flexibility decreased, and more hard and brittle structures were observed in biocomposite films. In addition, adding the reinforcement material to PVA triggered shear-thinning behavior, and the viscosity changed because of the interaction between the reinforcement and the matrix, and accordingly, the rheological properties were affected.
- The electrical measurement results showed that electrical conductivity increased as the amount of carbonized waste rubber increased. This increase in conductivity, particularly the significant 27.5% jump observed with the addition of 1% carbonized waste rubber, suggests a threshold value at which conductive networks within the composite become more efficient in facilitating electron transport.
- Since a smooth and homogeneous morphology was seen from the SEM images, it was concluded that PVA and carbonized waste rubber were compatible with each other.
- According to the nanoindentation hardness (H) and reduced elastic modulus results, it was observed that the addition of carbonized waste rubber caused an increase in hardness and a decrease in flexibility in the obtained biocomposite films, and this contributed to its distribution in the PVA matrix.

As a result, it can be concluded that biocomposite films obtained by increasing the electrical conductivity and hardness of PVA can be used in the electronics industry. The biocomposite films can be coated on various surfaces such as furniture, flooring materials, building components and electronical devices to obtain harder surfaces. Using waste tire rubbers in edit value end use areas may help to acquire circular economy.

## Figures and Tables

**Table 1:** Thermal properties of biocomposite films.

Sample	T5% (°C)	Max. Weight Loss (°C)	Char Yield (%)	Tm (°C)
PVA	203	372	5.51	225
PVA0.5CR	160	302	6.12	224
PVA1CR	175	309	4.07	223

**Table 2:** Conditions of the rheological test of biocomposite films.

Samples	Osc. Strain Sweep	Osc. Freq. Sweep
PVA	Eq = 1 min	Eq = 1 min
	Stress = 0.01–1000	ω = 0.01–100 rad/s
	f = 1 Hz	Strain = 1
	T = 230	T = 230
PVA0.5CR	Eq = 1 min	Eq = 1 min
	Stress = 0.01–1000	ω = 0.01–100 rad/s
	f = 1 Hz	Strain = 0.5
	T = 210	T = 210
PVA1CR	Eq = 1 min	Eq = 1 min
	Stress = 0.01–1000	ω = 0.01–100 rad/s
	f = 1 Hz	Strain = 0.1
	T = 210	T = 210

**Table 3:** Electrical conductivity measurements of biocomposite films at room temperature.

**Sample**	**I [A]**	**R Calculated**	**Length [mm]**	**Diameter [mm]**	**Section [m^2^]**	**Electrical Conductivity [S/m]**
PVA	1.81 × 10^−9^	552,486,187.8	0.33	22	0.00037	1572
PVA0.5CR	2.15 × 10^−9^	465,116,279.1	0.3	22	0.00037	1697
PVA1CR	2.38 × 10^−9^	420,168,067.2	0.32	22	0.00037	2004

## Data Availability

Data are contained within the article.
